# Abdominal Stent-Graft Treatment of Ascending Aortic Pseudoaneurysm Following Transcatheter Aortic Valve Implantation

**DOI:** 10.3390/medicina59010016

**Published:** 2022-12-21

**Authors:** Dimitrij Kuhelj, Črt Langel, Matjaž Bunc, Juš Kšela

**Affiliations:** 1 Institute of Radiology, University Medical Center Ljubljana, Zaloška 7, 1000 Ljubljana, Slovenia; 2 Faculty of Medicine, University of Ljubljana, Vrazov trg 2, 1000 Ljubljana, Slovenia; 3 Department of Cardiology, University Medical Center Ljubljana, Zaloška 7, 1000 Ljubljana, Slovenia; 4 Department of Cardiovascular Surgery, University Medical Center Ljubljana, Zaloška 7, 1000 Ljubljana, Slovenia

**Keywords:** stent graft, ascending aorta, valve-in-valve, TAVI, TEVAR

## Abstract

An ascending aortic pseudoaneurysm is a potentially lethal complication in aortic procedures. We present a hybrid approach using surgical innominate artery access and the endovascular insertion of an abdominal stent-graft extension to successfully treat a zone 0 ascending aortic pseudoaneurysm in a patient with a prior valve-in-valve transcatheter aortic valve implantation.

## 1. Introduction

Aortic pseudoaneurysms are a rare yet potentially lethal complication of many aortic procedures, including surgical aortic root replacement and transcatheter aortic valve implantation (TAVI) [1]. Such clinical scenarios are primarily treated by a re-do surgical procedure, predominately due to the fact that surgical treatment offers the best short- and long term survival rates. However, large retrosternal pseudoaneurysms, which are prone to rupture upon chest exploration, may favor minimally invasive approaches [2,3]. A prior valve-in-valve (ViV) TAVI presents an additional scarcely researched variable in patients with retrosternal ascending aortic pseudoaneurysms.

## 2. Case Presentation

**Figure 1:**
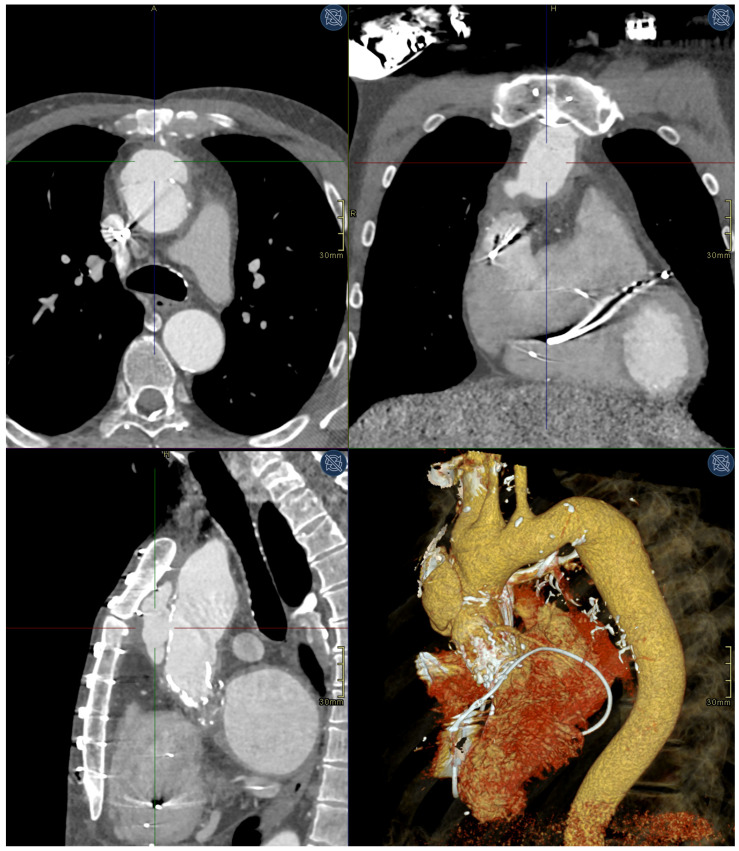
Preprocedural CTA showing a 58 × 55 × 30 mm pseudoaneurysm anteriorly in the ascending aorta. Clockwise from left bottom: sagittal plane arterial phase CTA, axial plane arterial phase CTA, coronal plane arterial phase CTA, volume rendering technique (VRT) 3D representation CTA.

A 59-year-old Caucasian male with an extensive cardiac history presented in 2021 with an asymptomatic 55 × 45 × 25 mm pseudoaneurysm of the Ishimaru zone 0 ascending aorta discovered by routine computed tomography angiography (CTA). The entry tear was located anteriorly at the distal anastomosis of the biological conduit. A follow-up CTA four months later showed the pseudoaneurysm size had increased to 58 × 55 × 30 mm (Figure 1).

His previous medical history included congenital aortic stenosis and a surgical aortic valve replacement (AVR) with mechanical prosthesis (Masters mechanical valve, Abbott, Abbott Park, IL, USA) performed in 1992, followed firstly by a re-do surgical AVR with biological prosthesis (Trifecta aortic valve, Abbott, Abbott Park, IL, USA) and the replacement of the ascending aorta (Vascutek Gelseal, Terumo Aortic, Inchinnan, UK) in 2014, due to a prosthetic valve re-stenosis and an ascending aorta aneurysm; secondly, by a re-re-do surgical Bentall procedure replacing the bioprosthetic valve, aortic root and aortic graft with biological conduit (BioIntegral No-React BioConduit, BioIntegral Surgical, Mississauga, ON, Canada) in 2015, due to bioprosthetic valve and vascular graft endocarditis; thirdly, by a re-re-re-do AVR using sutureless biological prosthesis (PercevalTM, Corcym, Saluggia, Italy) in 2017, due to repeated bioprosthetic valve endocarditis; and finally, by a rescue extracorporeal membrane oxygenation (ECMO)-supported ViV TAVI in 2019, due to bioprosthetic valve degeneration and deteriorating cardiogenic shock. After the last procedure, he completed the protocol for inclusion into the heart transplantation list. An implantable cardiac resynchronization therapy defibrillator (CRT-D) was additionally implanted. After rehabilitation, he was released into home care with normal kidney function and a New York Heart Association (NYHA) II classification. His latest echocardiography showed left ventricular dilatation (end-diastolic diameter (EDD) 5.8 cm) with a mildly reduced left ventricular ejection fraction (LVEF 46%), good functioning of the prosthetic valve (mean transprosthetic gradient 20 mmHg, aortic valve area index (AVA) 2.01 cm^2^, minimal paravalvular leak) and mild pulmonary hypertension (systolic pulmonary artery pressure (sPAP) 29 mmHg).

After re-admission due to the asymptomatic ascending aortic pseudoaneurysm, a multidisciplinary team discussed the case and decided not to perform another open surgery but instead opt for a less invasive but technically extremely demanding hybrid thoracic endovascular aortic repair (TEVAR).

Since the intended landing zone measured 35 mm in diameter and less than 100 mm in length, no commercially available TEVAR device could be deployed. Rather, a 38 × 50 mm abdominal stent-graft (SG) extension (JOTEC, CryoLife, Hechingen, Germany) was selected. As the delivery catheter of this particular device is relatively short, precluding transfemoral delivery, access to the distal portion of the innominate artery (IA) was established surgically by the right supraclavicular skin incision. Digital subtraction angiography (DSA) of the thoracic aorta and branches with coronary angiograms was performed to confirm anatomical relations. In addition to the fluoroscopy, a transesophageal echocardiogram (TEE) was used to guide the procedure, lowering the exposure to ionizing radiation and contrast media.

**Figure 2:**
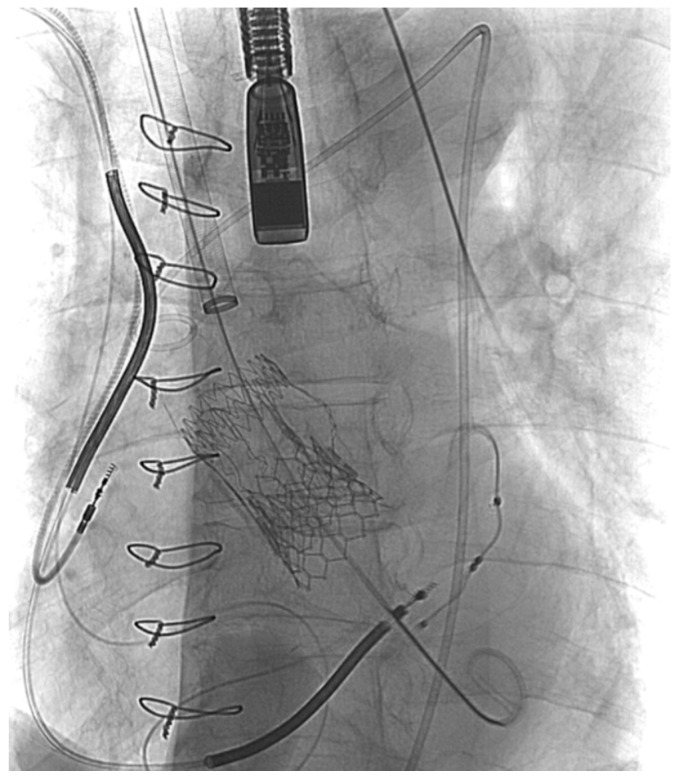
TEVAR fluoroscopy: TAVI and Bentall valves. Note also a vascular sheath, a stiff guidewire, and a TEE transducer.

**Figure 3:**
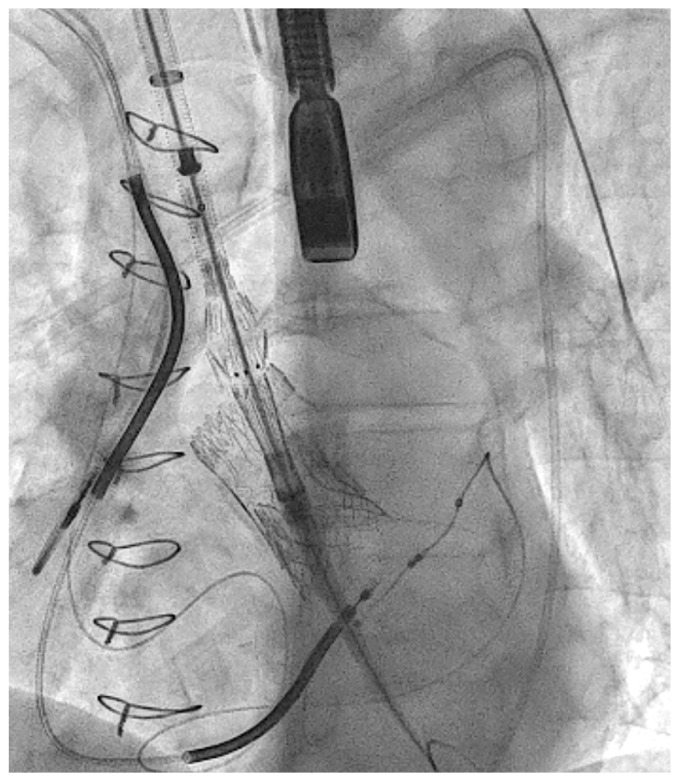
TEVAR fluoroscopy: TEVAR fluoroscopy 2: initial implantation of JOTEC 38 × 50 mm abdominal SG extension.

**Figure 4:**
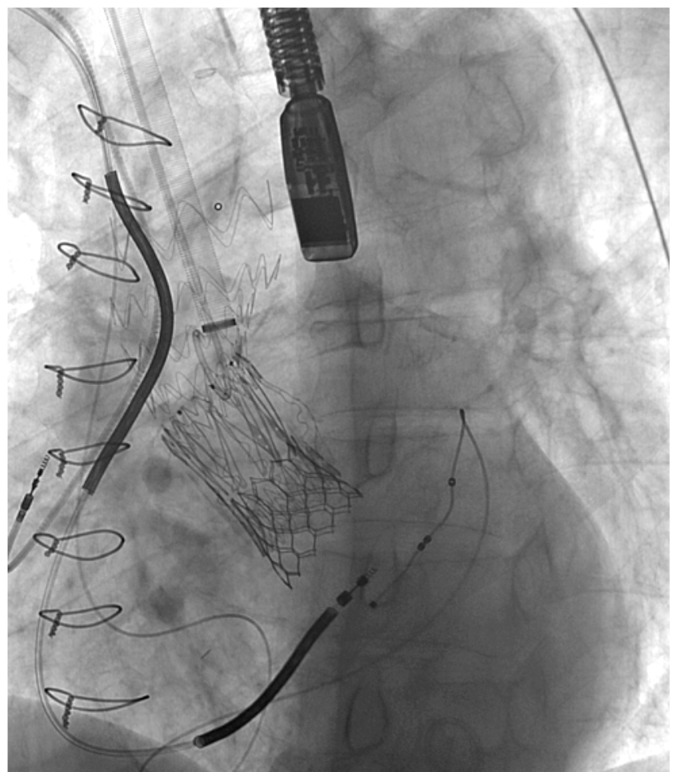
TEVAR fluoroscopy 3: final result with deployed JOTEC SG.

After the distal portion of the IA was surgically dissected, two double armed 4-0 Prolene purse-string sutures were placed on the anterior portion of the IA, and a stiff guidewire was inserted through it. Next, the stiff guidewire was navigated carefully through the TAVI prosthesis in order not to damage it (Figure 2). The SG was inserted with its conventional distal part positioned toward the left ventricle, and deployed in zone 0 (Figure 3 and Figure 4).

**Figure 5:**
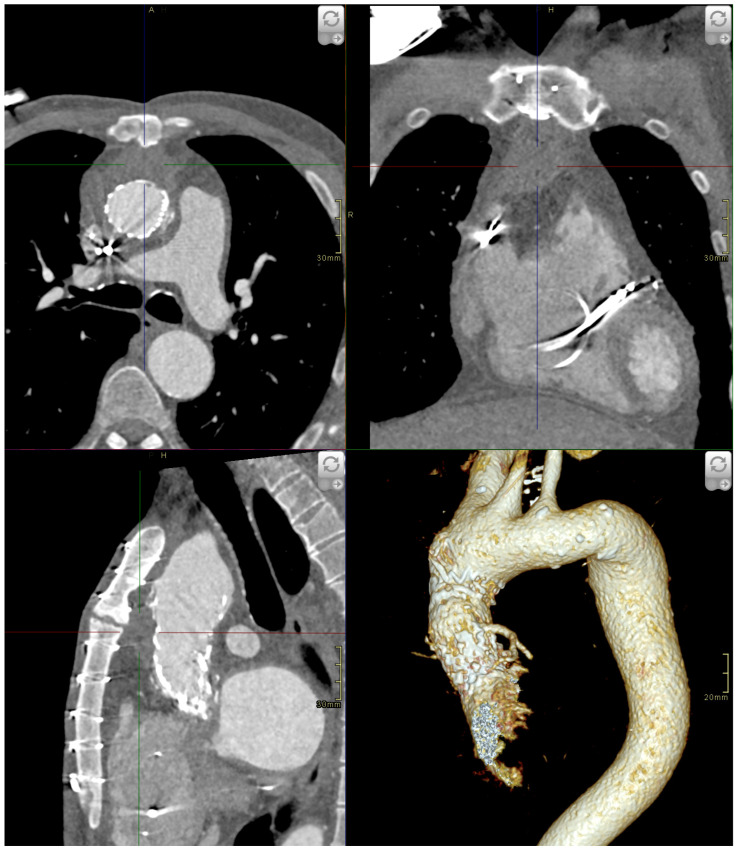
Two months’ follow-up CTA showing complete exclusion of the pseudoaneurysm. Clockwise from left bottom: sagittal plane arterial phase CTA, axial plane arterial phase CTA, coronal plane arterial phase CTA, volume rendering technique (VRT) 3D representation CTA.

Control angiograms confirmed an adequate device position, a minimal distal type 1 endoleak with a slight residual filling of the pseudoaneurysm, patent arch branch vessels, functional TAVI, and patent coronary arteries with catheter selective accessibility. The IA access site was closed surgically, while the left femoral access was closed with Perclose Proglide (Abbott Vascular, Redwood City, CA, USA). There were no periprocedural complications. A dose of 7000 IU of heparin was administered during the procedure. After the procedure, the patient continued taking 100 mg of acetylsalicylic acid daily. A follow-up CTA one week later showed the pseudoaneurysm lumen size decreasing to 25 × 15 × 25 mm. Another CTA two months later showed complete regression of the pseudoaneurysm sac with normal aortic luminal flow (Figure 5). At that point, the acetylsalicylic acid was discontinued and the patient commenced with a daily 220 mg dabigatran etexilate anticoagulation therapy, due to preexisting atrial fibrillation. The patient remained under close observation and reported no complications at the one-year follow-up. CTA one year after the procedure revealed continued complete pseudoaneurysm exclusion and a decrease in mural thrombus thickness from 25 to 12 mm.

## 3. Discussion

An ascending aortic pseudoaneurysm is a known major complication of both surgical and endovascular aortic procedures, typically arising in months to years after the primary procedure. While they occur in up to 3% of patients after successful aortic surgery, data on TAVI-related incidence of pseudoaneurysms are lacking [4]. The most common location is the distal anastomosis on the ascending aorta [5]—as was the case with our patient. The most common clinical presentation includes dyspnea (44%), chest pain (39%), and fever (32%). However, they may be asymptomatic in 21% of cases [5] or, rarely, cause pulsatile sternal swelling [6].

The possible ascending aortic pseudoaneurysm etiology in the setting of prior aortic surgery and/or endovascular procedures includes graft infection, nonoptimal Bentall suture line tension between the artificial graft and native aorta [7], persistent perigraft bleeding [7], excessive use of biological glue [8,9], and excessive mechanical forces acting on the vessel wall during TAVI [10]. Our patient had a history of previous graft infections (those grafts had been surgically replaced), but the most recent pre-TAVI workup indicated no fever or inflammatory marker elevation. No balloon overinflation had occurred during the TAVI procedure, but the ascending aortic wall could have been injured mechanically by the guide wire manipulation during the TAVI procedure. Careful wire selection can lower the probability of iatrogenic vessel wall injury, and wires with pre-shaped distal ends tend to reduce the risk of injuring vessels or ventricles [11]. The risk of vascular damage can also be mitigated by using guide wires of lesser stiffness—unfortunately, the use of stiffer guidewires is sometimes unavoidable to achieve technical procedural success [12]. Taking into account the location of the pseudoaneurysm that originated at the distal anastomotic suture line, it is unlikely to have been caused by the in situ TAVI prosthesis—a case of more proximal ViV-TAVI-related ascending aortic pseudoaneurysm has been reported, though [10]. All factors considered, the exact cause of the pseudoaneurysm occurrence in our case cannot be pinpointed with certainty.

As is the case with other pseudoaneurysms, an ascending aortic pseudoaneurysm can expand and potentially lead to complications, such as compression or erosion of the nearby structures, fistula formation, bleeding, or, most critically, a rupture with the resulting massive bleeding and possible exsanguination [13,14]. Pseudoaneurysms can also become a nidus of recurrent infection or embolism [15]. No definitive factors of pseudoaneurysm expansion rates have so far been found [16].

The ascending aortic pseudoaneurysm standard treatment option is surgical excision, requiring cardiopulmonary bypass and carrying significant risks. According to research performed by Villavicencio et al. that included 57 patients with surgical ascending aortic pseudoaneurysm repairs, a pseudoaneurysm with a size greater than 55 mm, NYHA functional class III or IV, and urgent operation were identified as predictors of hemorrhage during a re-do sternotomy. There was also a 7% operative mortality, with obesity and ejection fraction (EF) of 35% or lower being the predictors of operative death [5]. Other research found the stroke rate in ascending aortic surgery to be 2.0–4.0% [17,18].

In our case, the pseudoaneurysm diameter was 58 mm and the patient had no other known risk factors for hemorrhage or mortality during re-do surgery. He had, however, multiple previous surgical aortic procedures, some complicated by mediastinitis, and a ViV TAVI, all of which present possible risk variables not previously evaluated in large cohort studies. TEVAR was thus favored, but there is risk associated with endovascular procedures, too. The principal complications in ascending TEVAR are premature stent deployment causing aortic insufficiency or myocardial infarction, stent distal migration with IA occlusion causing stroke, left ventricle perforation, cardiac tamponade, injury and dissection of the aortic root, residual aortic dissection, and endoleak [19]. A systematic review of non-dissected ascending aorta disease by Wang et al. revealed an endoleak rate of 13.4%, stroke rate of 4.8%, and non-ST-elevation myocardial infarction of 1.5% [20].

As mentioned, the short distance between the pseudoaneurysm entry tear and the IA branch-off presented a challenge for SG selection. Several techniques have been described for ascending aortic pathologies with landing zones too short to deploy a conventional SG, including fenestrated TEVAR [21], IA debranching and Ishimaru zone 0 TEVAR with a single chimney to the IA [3], closing the pseudoaneurysm using an Amplatz Type II or Type III vascular plug (AVP) only, or first coil embolizing the pseudoaneurysm followed by AVP insertion [22], and even a novel Endo-Bentall procedure [23]. In our case, the short landing zone challenge was overcome by selecting an off-the-shelf SG of the appropriate dimensions and by careful deployment preplanning to ensure continued patency of the IA.

We faced several other challenges. The particular SG uses a short 20F delivery system, eliminating the possibility of a transfemoral approach. The patient’s slightly dilated IA of sufficient caliber to accommodate the delivery system was thus chosen for surgical vascular access.

Another concern was minimizing the risk of interaction between the delivery device and the TAVI prosthesis. Many cautious attempts were made before successfully inserting the guidewire through the center of the TAVI rather than its bare springs. The selected 38 × 50 JOTEC abdominal SG extension features a short nose cone, reducing interactions and possible damage to the aortic valve during insertion.

The stable positioning of the delivery system was also a challenge. To counter blood flow pulsatile forces and increase overall delivery stability, the systolic blood pressure was lowered to 50 mmHg using overdrive cardiac pacing; a stiff guidewire with a flexible tip was positioned deep inside the left ventricle, and the delivery sheath was wedged between the right lateral IA wall and the left lateral ascending aorta wall.

During the procedure, a minimal distal type 1 endoleak was observed and considered in advance since less than 10% oversizing was planned due to the limited diameters of the SG extensions. A type 1 endoleak is a known TEVAR complication, occurring in about 13% of procedures [24]. Factors that predict endoleak include larger aneurysm size, the length of the aorta treated by stent grafts, an increasing number of stents used, and male sex [24]. Considering the small rate of leakage in our case, it was decided not to treat it with ballooning during the same session. Indeed, the follow-up CTA indicated no residual endoleak.

As a side note, it is interesting that the ascending aortic branch-free anatomy precludes the occurrence of type II and, in cases where only a single graft is inserted, also of type III endoleaks—hence, endoleaks in ascending TEVAR mainly comprise type Ia and Ib [19].

A complete thrombosis of the pseudoaneurysm sac was observed on the two-month and one-year follow-up CTAs, and our patient remained in good health—An outcome consistent with a previously published research finding on TEVAR in high-risk patients with ascending aortic pathology, showing to have high survival rates of 86% at 30 days, 80% at 1 year, and 75% at 5 years [2].

## 4. Conclusions

This case demonstrates that the IA approach TEVAR, using an off-the-shelf abdominal SG extension, may be a safe, feasible and efficacious method to treat post-ViV TAVI ascending aortic pseudoaneurysms. The technical aspects specific to ascending aortic endovascular procedures in general and with regard to previously inserted TAVI endoprosthesis should be studied and prepared for in advance.

## Data Availability

Not applicable.
